# Hoarseness due to subcutaneous emphysema: a rare presentation of diverticular perforation

**DOI:** 10.1093/jscr/rjad566

**Published:** 2024-03-14

**Authors:** Sydney L Bormann, Rebekah Wood, Jenny M Guido

**Affiliations:** Department of Surgery, University of South Dakota Sanford School of Medicine, Sioux Falls, SD 57105, United States; Department of Surgery, University of South Dakota Sanford School of Medicine, Sioux Falls, SD 57105, United States; Department of Surgery, University of South Dakota Sanford School of Medicine, Sioux Falls, SD 57105, United States

**Keywords:** perforated diverticulitis, Addison’s disease, subcutaneous emphysema

## Abstract

Pneumomediastinum and subcutaneous emphysema usually result from alveolar rupture and rarely from colonic perforation. Although steroid use has been shown to increase the risk of complicated diverticulitis, there is limited data on the role Addison’s disease may play in the development of colonic perforation. We present a rare case of a patient with Addison’s disease who presented with hoarseness and was found to have massive subcutaneous emphysema, pneumomediastinum, and pneumoretroperitoneum secondary to complicated diverticulitis.

## Introduction

Pneumomediastinum and subcutaneous cervical emphysema usually occur due to spontaneous alveolar rupture with subsequent dissection into the mediastinum and cervical soft tissues from the retroperitoneum [1]. Less commonly, pneumomediastinum and subcutaneous emphysema can be caused by colonic perforation due to diverticulitis, toxic megacolon, colonoscopy, endoscopy, and polypectomy [2]. Clinical findings of subcutaneous emphysema include dyspnea as well as swelling, crepitus, and pain over the neck and chest [3]. Rarely, subcutaneous emphysema may present with hoarseness and vocal changes [4, 5]. Delay in diagnosis of retroperitoneal colonic perforation resulting in pneumomediastinum and subcutaneous emphysema can result in significant morbidity and mortality [2]. We present a rare case of a patient with Addison’s disease who presented with hoarseness and underwent workup with diagnosis of massive retroperitoneal, mediastinal, and cervical subcutaneous emphysema secondary to perforated diverticulitis. He subsequently proceeded to the operating room for Hartmann’s end colostomy.

## Case report

A 90-year-old male presented to the emergency department with a 2-day history of hoarseness, shortness of breath, chest tightness, and generalized weakness. Upon further interrogation, he was also experiencing fever and mild abdominal discomfort. His past medical history was significant for Addison’s disease, which was previously well controlled with hydrocortisone 20 mg three times daily and fludrocortisone 0.05 mg once daily. He had no prior history of Addisonian crises requiring hospital admission.

**Figure 1:**
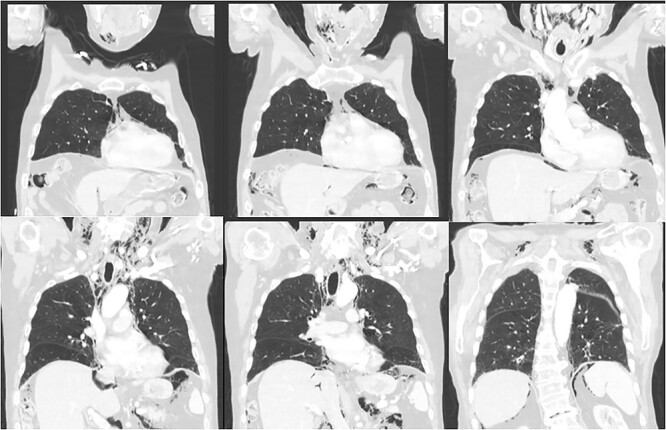
CT scan of the chest demonstrating subcutaneous emphysema and extensive free gas in the mediastinum and retroperitoneum.

**Figure 2:**
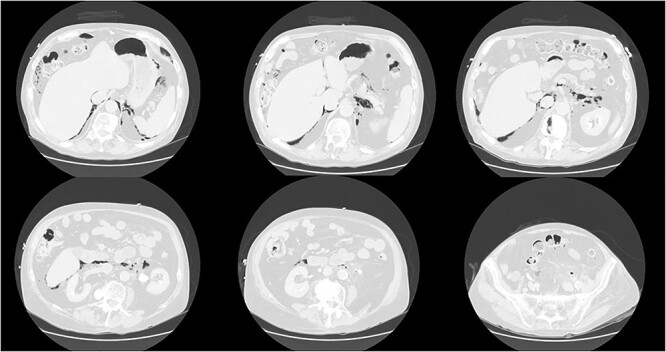
CT scan of the abdomen and pelvis demonstrating perforation of the sigmoid colon and free gas in the retroperitoneum and mediastinum.

His vitals were stable on presentation. Physical exam was significant for weakness, tachypnea, and wheezing. He had no appreciable subcutaneous emphysema on clinical exam. Workup in the emergency department was remarkable for increased white blood cell count (17.0 K/uL). EKG showed no acute changes and cardiac enzymes were within normal limits. Computed tomography (CT) of the chest, abdomen, and pelvis with contrast was obtained, demonstrating acute sigmoid diverticulitis with extensive retroperitoneal air originating next to the sigmoid colon and multiple small collections of fluid. The pneumoretroperitoneum was continuous with pneumomediastinum and cervical emphysema (Figs 1 and 2). He was diagnosed with perforated diverticulitis and operative intervention was recommended.

Emergent exploratory laparotomy was performed confirming acute retroperitoneal perforation of sigmoid diverticulitis. He underwent sigmoid colectomy with end colostomy formation. He initially recovered well postoperatively with stress dose steroid therapy of hydrocortisone 50 mg six times daily. The dosage was tapered down to his home dose of hydrocortisone 20 mg three times daily by postoperative day 4. On postoperative day 5, the patient had an episode of nausea and emesis which prevented him from taking PO hydrocortisone and he was instead given a 4 mg dose of IV methylprednisolone. Later that day, he suffered from an adrenal crisis with symptoms including tachycardia, hypotension, and weakness and required transfer to the intensive care unit. The hydrocortisone dose was increased to 20 mg four times daily with improvement in his symptoms. His hospital course was further complicated by an abdominal abscess on postoperative day 11, and he underwent CT-guided placement of a percutaneous drainage tube. On postoperative day 19, he was discharged to a swing bed facility.

## Discussion

Addison’s disease is a rare disorder most commonly due to autoimmune causes. Common clinical symptoms including weakness, fatigue, weight loss, nausea, and vomiting, are due to deficient production of glucocorticoids, mineralocorticoids, and androgens. Due to the wide variation in symptoms and clinical presentations, Addison’s disease may mask the symptoms of other co-existing conditions [6]. The delayed presentation of the patient in this case study may have been influenced by existing symptoms of Addison’s disease and concomitant steroid use. Peritonitis from pneumoperitoneum or abscess formation commonly cause patients to seek medical evaluation for their abdominal pain. In this case study, the patient’s absence of severe abdominal pain is an unusual presentation [7]. To our knowledge, there is no existing literature suggesting Addison’s disease as a risk factor for colonic perforation; however, literature supports steroid use as a potential risk for complicated diverticulitis and ensuing complications postoperatively [8–10]. In patients receiving steroids, abdominal signs are sometimes unreliable, making diagnosis difficult. One case study reports a patient with rheumatoid arthritis receiving prednisolone who presented with sigmoid perforation resulting in mediastinal emphysema [2]. In patients receiving prednisone at 20 mg/day or more, clinical symptoms may not be noticeable even in cases of peritonitis resulting from perforation or abscess [11]. In patients taking steroids who presented with bowel perforation, mortality rate of patients receiving a dose of 20 mg/day or less was 13.3%, while mortality in those receiving a dose greater than 20 mg/day was 85.1% [12].

Pneumomediastinum and subcutaneous emphysema are rare signs of complicated diverticulitis which may delay diagnosis. A literature review demonstrated that 20 cases of mediastinal emphysema following colonic perforation have been reported [2]. Typical findings of subcutaneous emphysema include swelling and crepitus over the neck and chest, neck and chest pain, and dyspnea [3]. The initial symptoms of hoarseness and weakness are an atypical presentation of perforated diverticulitis, which made diagnosis challenging. There are case reports of pneumomediastinum presenting with hoarseness and alteration in voice [4, 5]; however, to our knowledge there are no reports of perforated sigmoid diverticulitis presenting with hoarseness. Patients presenting with cervical subcutaneous emphysema without a thoracic etiology require further assessment for gastrointestinal causes. Prompt surgical treatment is required when complicated diverticulitis is the cause.
